# The integration of quality improvement and implementation science methods and frameworks in healthcare: a systematic review

**DOI:** 10.1186/s12913-025-12730-9

**Published:** 2025-04-16

**Authors:** Mia Bierbaum, Stephanie Best, Sharon Williams, Zoe Fehlberg, Susan Hillier, Louise A. Ellis, Angie Goodrich, Robert Padbury, Peter Hibbert

**Affiliations:** 1 Australian Institute of Health Innovation, Macquarie University, Sydney, Australia; 2 University of South Australia, Adelaide, Australia; 3 University of Melbourne, Melbourne, Australia; 4 Swansea University, Swansea, Wales; 5 Southern Adelaide Local Health Network, Adelaide, Australia; 6 Flinders University, Adelaide, Australia

**Keywords:** Implementation science, Quality improvement, Systematic review, Narrative synthesis, Tertiary healthcare, Hospitals, Integration, Quality of healthcare

## Abstract

**Objectives:**

Quality Improvement (QI) and Implementation Science (IS) are both frequently utilised in health research. Little is known about how they are integrated within studies, and whether combined they add value. This systematic review sought to investigate how QI and IS theories and strategies are integrated within healthcare-based studies.

**Methods:**

A systematic search was conducted across five databases. Duplicates, studies published prior to 2014, systematic and scoping reviews, and study protocols were removed. The retrieved title abstracts were screened, and the full texts of eligible studies were reviewed in pairs using Covidence software. Of the included studies, data were extracted using a predefined template, and studies were critically appraised using the QI Minimum Quality Criteria Set. Frequency analysis of the use of QI or IS tools was conducted, as well as a narrative analysis of the integration of QI and IS in each study.

**Results:**

The database search returned 3,407 title abstracts, of which 1,618 were screened. Assessment for eligibility resulted in the identification of 149 studies, of which the full texts were reviewed, and 12 studies included in the final analysis. These 12 studies integrated QI and IS methods to implement an intervention in tertiary healthcare. The Plan-Do-Study-Act (PDSA) cycle was the most frequently used QI tool and the Theoretical Domains Framework, Behaviour Change Wheel (including Capabilities, Opportunity and Motivation) and the Consolidated Framework for Implementation Research were the most frequently used IS frameworks.

**Conclusion:**

The study highlights a lack of consistent terminology across the QI and IS fields, as well as opportunities for greater integration of the two fields to enhance study design, implementation and sustainability, and to improve healthcare performance.

**Supplementary Information:**

The online version contains supplementary material available at 10.1186/s12913-025-12730-9.

## Introduction

Quality Improvement (QI) and Implementation Science (IS) share a common goal of improving quality in healthcare. While there are similarities across both disciplines their histories and modus operandi vary. There are many definitions of QI; however, the most commonly quoted is the Academy of Medical Royal Colleges definition which suggests moving away from a single method or set of tools, and to think of QI as a systematic continuous approach to problem solving in healthcare with the aim of improving service provision and provide better quality of care and ultimately outcomes for patients [1]. QI has a long track record grounded in healthcare and QI studies commonly focus on identifying specific local and context specific challenges in a health system at the provider, clinic or patient level [2]. Adopting a wide range of assessment and measurement methods, many of which have been adapted from business, such as Lean and Six Sigma [3], QI identifies the locus of a health system challenge to design and test setting specific interventions [1, 4].

Implementation Science (IS), “*the scientific study of methods to promote the systematic uptake of research findings and other evidence-based practices into routine practice, and, hence, to improve the quality and effectiveness of health services*” ([5], p1) has a more recent history originating in rural sociology [6]. IS draws on theories, models and frameworks from behaviour change and social psychology to design and test implementation strategies to support uptake or adoption of evidence-based interventions. IS explicitly considers the role of creating generalisable evidence that can be used in other settings beyond the immediate context. Both QI and IS share a common ambition, attention to process and outcomes with some common methods. A recent review has compared and contrasted studies using QI or IS methods and approaches to achieve practice change in cancer care, highlighting potential for synergies to reduce duplication and enhance care outcomes [7].

Despite having two complementary approaches to improving quality in healthcare, endeavours to bring the two disciplines together have been somewhat limited and the use of terminology in both improvement and implementation research has been unclear. While much of the terminology of QI and IS appears at face value to be straightforward, there is concern in the field that the underuse and misuse of theories, models and frameworks presents as a challenge to growing the evidence base in improvement and implementation research [8].

The aim of this review was to understand the way in which QI and IS theories and strategies are integrated within healthcare-based studies. To the best of our knowledge this synthesis has not been previously undertaken across healthcare services.

## Methods

The protocol for this systematic review was registered on Prospero (2024) (registration no. CRD42024553059). The review follows the Preferred Reporting Items for Systematic Reviews and Meta- Analyses (PRISMA) guidelines [9] (Supplementary file 1. PRISMA checklist). This systematic review aimed to answer the research question: “How do hospital-based studies integrate QI and IS methods, theories, tools and strategies?”.

### Search strategy

Title abstract searches were conducted across 5 databases (Ovid Embase, Ovid MEDLINE, Ovid Emcare CINAHL, Web of Science) in June 2024. Librarian advice and support was sought to refine the search strategy (Supplementary file 2 Medline search strategy). The search included studies from 2014 to June 2024 using the Embase search string:

- (exp Implementation Science/or exp "diffusion of innovation"/or ("The Consolidated Framework for Implementation Research" or "Theoretical domains framework" or "Reach effectiveness adoption implementation Maintenance" or "RE AIM" or "The Knowledge-to-Action Framework" or "Diffusion of Innovation* Theory" or "Implementation climate scale" or "Com-b" or "reach, effectiveness, adoption, implementation, and maintenance framework").ti,ab,kf.) AND (exp Quality Improvement/or total quality management/or exp "Root Cause Analysis"/or ("Quality Improvement" or "total quality management" or "Continuous Improvement" or "Improvement science" or "lean methodology" or "Lean management" or "Plan-Do-Study-Act cycle" or "PDSA" or RCA or "Root cause analys*" or Kaizen or "Six sigma" or "six sigma methodology" or "Institute for Healthcare Improvement Model for Improvement" or "Theory of constraint*").ti,ab,kf.).

### Inclusion and exclusion criteria

#### Inclusion criteria

Studies were included if they were: based in a hospital setting; about a healthcare condition/healthcare professionals; and Integrated QI methods, theories and frameworks with IS theories, models or frameworks within the implementation of an intervention. Studies must have stated they used QI methods and have provided evidence of using QI methods/models/theories/frameworks. Studies must have also stated they used IS methods and have provided evidence of using IS methods/models/theories/frameworks. Studies must have the full text of an empirical study available and been published in a peer reviewed journal, between 2014 to June 2024 in English. Studies were limited to tertiary hospital settings to enable comparison between similar settings, while studies published since 2014 were included to review contemporary literature reflecting current trends in methodology use and integration.

#### Exclusion criteria

Studies were excluded if they: used IS theory/models/frameworks for diagnostic purposes (for example, using IS theory to identify barriers and facilitators to the implementation of an intervention, without reporting the application of those findings in the implementation of the intervention). Review articles identified by the search were reviewed for snowballing of additional studies but otherwise excluded from analysis.

### Study selection

Titles and abstracts were downloaded from databases and screened against the inclusion criteria. Titles were divided and screened by six pairs of reviewers: MB paired with PH, SB, SW, SH, ZF and LAE using Covidence software [10]. Full texts of the abstracts which met the inclusion criteria were then retrieved, divided and reviewed by four pairs: MB paired with SB, SW, ZF and SH, again using Covidence software. All disagreements were discussed as a group and resolved through team consensus. MB reviewed all titles and full texts to increase consistency and rigour.

### Data extraction

Data were extracted from each eligible study and recorded in a purpose designed Excel spreadsheet. Data included: citation; the location of the study (country and setting e.g., hospital); the study design; the population studied (including staff or patients); data collection methods; QI change initiative; study aim; IS elements identified in the study; QI elements identified in the study; and the described process of integration of QI and IS elements. We also extracted whether ethics approval was sought or received, and whether studies described following a reporting guideline. Data were extracted from the included studies by MB and verified by one co-author (ZF). Disagreements or discrepancies were resolved by team consensus.

### Quality appraisal

The Quality Improvement Minimum Quality Criteria Set (QI-MQCS) was used to critically appraise the reporting of the included studies. This tool guides the assessment of each study across 16 domains, or reporting standards, to guide whether the minimum criteria were met for each study. For a study to be considered high quality, a minimum of 14 or more criteria must be reported [11]. This tool was deemed appropriate given all included studies identified as a QI project.

### Data analysis and synthesis

After extracting key data, a frequency count of each QI or IS theory/tool/method used was conducted along with a narrative synthesis [12] of the methods of QI and IS integration in the included studies. This narrative analysis identified why each tool/method/theory was used, for example, to *identify barriers and facilitators (B&Fs) to implementation*. This process of categorising the use of each tool allowed the inductive identification of key study phases, in which each of the tools and methods were used. These study phases were reviewed and defined by five reviewers (MB, PH, SW, SB and ZF) and agreed upon through team consensus. The frequency count of the use of QI or IS methods/tools/theories was then used to identify how frequently QI or IS methods/tools/theories were used across the different study phases. A greater explanation of the analysis can be seen in Supplementary file 3. The key inductively identified study phases included:

- *The System diagnostic phase*, which we defined as an assessment of the extent and/or nature of an issue being targeted to improve performance or outcomes, and identification of B&Fs to implementation. This included: QI methods/tools/theories used to identify B&Fs to implementation (e.g., Process Mapping, Fishbone diagram/Cause and effect diagram, Pareto chart, Force field analysis, Impact effort matrix, and histograms), and IS tools/theories used to identify B&Fs to implementation (e.g., COM-B, TDF, CFIR).
- *The Intervention design phase* which typically involves the design, development and refinement of an intervention. This included: QI/IS methods/tools/theories used to inform the QI design.
- *The Implementation of intervention phase* which typically included intervention testing and embedded strategies to implement the intervention. This included: QI tools that guided implementation strategies (e.g., Plan, Do, Study, Act (PDSA), Audit and & Feedback (A&F), and Champions), IS tools/theories that guided implementation strategies, and Feasibility and useability testing.
- The *Scale/spread or sustainability phase* which included scale up of the intervention to a larger or different team or setting with consideration of ongoing maintenance of the implementation of the intervention. This included IS tools/theories used to determine whether it was appropriate to upscale the intervention across the organisation.
- As well as these four phases, *Methodology* (which included methodologies that were applied across the entire span of the study, such as Lean six sigma), and *Measurement tools* such as Control charts and Run charts were included in the analysis.

## Results

### Study selection

**Fig. 1:**
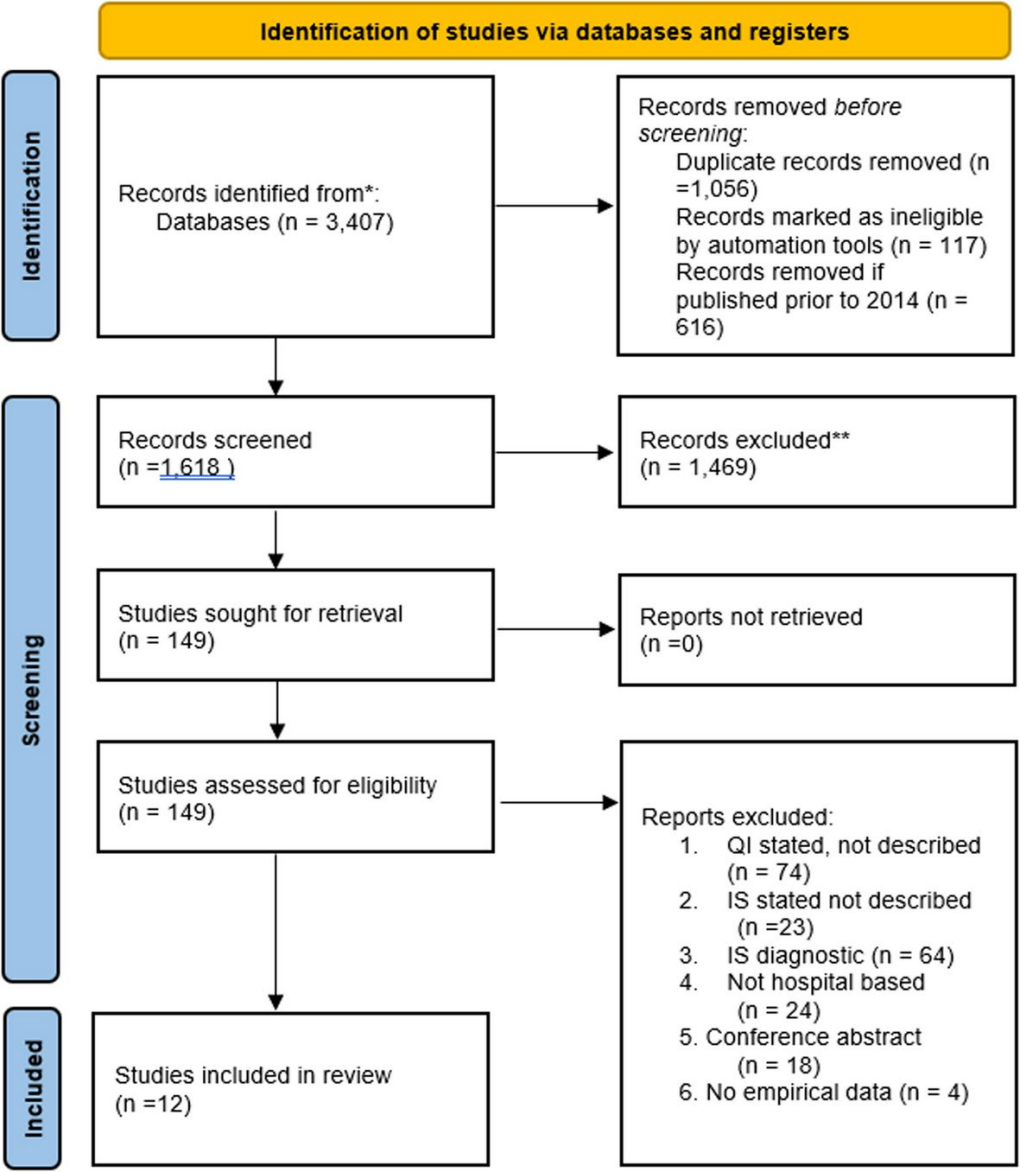
PRISMA flow diagram

The five-database search returned 3,407 titles (Ovid Medline (*n* = 1,384), Ovid Embase (*n* = 1,018), Ovid Emcare (*n* = 406), CINAHL (*n* = 137) and Web of Science (*n* = 462). Duplicates were removed (*n* = 1,056) as well as studies published prior to 2014 (*n* = 616), and systematic reviews, scoping reviews and study protocols (*n* = 117). A total of 1,618 title abstracts were then screened in Covidence software, resulting in 1,469 studies being excluded that did not meet the inclusion criteria. Full text screening was undertaken on the remaining 149 studies, and a further 137 studies were excluded. A total of 12 manuscripts met the inclusion criteria and were included in the final review [13–24] (Fig. 1). No additional studies were identified during the snowball analysis of included studies.

The six main reasons for exclusion were: 1) If QI was stated but not described, which typically included studies that described the project as a QI project, but did not clearly describe QI methods or tools (*n* = 74); 2) If IS was stated but not described, which typically included studies that described the project integrating IS elements or theories, but did not clearly describe the IS theory or methods (*n* = 23); 3) If IS was used for diagnostic purposes, which typically included studies that used an IS theory, framework or model to inform their evaluation of barriers and facilitators to implementation, but did not report the application of those findings (*n* = 64); 4) if the study was not hospital- or tertiary care- based (*n* = 24); 5) the full text search identified that the title referred to a conference abstract, preprint or thesis (*n* = 18), and; 6) No empirical data were reported (including reviews) (*n* = 4), noting that some studies had multiple reasons for exclusion.

The interrater reliability between pairs was initially poor, with Cohen’s Kappa scores [25] ranging from slight agreement (0.10–0.20) to fair agreement (0.21–0.40), reflecting the complexity of this review. As a result, all disagreements were discussed as a team in regular team meetings, and consensus reached as to whether a manuscript would be included or excluded, and why.

### Critical appraisal

The QI-MQCS tool was used to critically appraise the 12 included studies. Only one quarter of studies (25%) (*n* = 3) [13, 20, 24] met the QI-MQCS minimum standard for reporting with a minimum score of 14/16 QI criteria [11] (Supplementary file 4). The mean QI-MQCS quality score was 11.8 (95% CI 10.97–12.70). All studies reported the following domains: Organisational motivation, Intervention rationale, Intervention description, Implementation, Data source, Timing, Limitations. The domains that were least often reported included: Spread (*n* = 3), Health outcomes (*n* = 3), Study design (*n* = 4), Penetration/Reach (*n* = 7), Sustainability (*n* = 7), Comparator (*n* = 7), Adherence/Fidelity (*n* = 8), Organisational readiness (*n* = 10), Organisational characteristics (*n* = 10).

### Study characteristics

#### Study design

Of the 12 included studies, over half described their study as a QI study without explicitly reporting a study design or methodology [14–16, 19, 22–24]. Five studies provided details about their study design, describing their studies as a staggered, pre-post quasi-experimental implementation study [13], implementation research [17], a sequential explanatory mixed methods study [18], participatory design methodology [20], and a participatory research study [21].

#### Study setting and topics

All studies were conducted in hospital settings, most commonly within the United States of America (USA) (*n* = 4), Canada (*n* = 2) (with an additional study potentially based in Canada, although it was not explicitly described [19]), the United Kingdom (UK) (*n* = 2), Brazil (*n* = 1), Ghana (*n* = 1), and Uganda (*n* = 1). The QI project topics were mostly heterogenous. Two studies were focused on reducing sepsis, one in a Neonatal Intensive Care Unit (NICU) [16], and the other in adult patients [22], and two studies were related to improving the appropriate use of laboratory tests, one in the Emergency Department (ED) [23] and one specifically reducing Blood Urea Nitrogen (BUN) ordering [18]. Other studies were focused on enhancing vital sign collection [13], developing a virtual cardiac rehabilitation program [14], developing a standardised post-fall debrief tool [15], implementing a screening tool to improve pain management referrals [17], improving SpO2 maintenance in NICU [19], developing an individualised performance data dashboard for clinicians [20], developing a care protocol for premature newborns in their first hour of life [21], and introducing an intradialytic exercise program for haemodialysis patients [24] (Table 1).

**Table 1:** Characteristics of included studies

Study	QI and study aim	Country	Setting	Participants	Patients of QI Int	Data collection methods	Study type as described	QI tools	IS tools	Ethics	Reporting guideline	Integration of QI and IS
Cummings [13]	Develop, implement and evaluate the QI intervention to improve diagnosis of illness through enhanced vital sign collection	Uganda	3 hospitals and 1 inpatient health facility	Hospital HCPs	Hospital-ised patients, 14 years +	Survey, Obs, FGs, MRR, activity mapping	Staggered, pre-post quasi-experimental impl. study	PDSA, activity mapping of the patient flow practices, A&F, champions	COM-B	Ap	ND	1. IS (COM-B) informed QI Int. design 2. IS (COM-B) identified B&Fs to QI Int. impl 3. B&Fs and QI (activity mapping, PDSA, A&F, champions) informed modification of QI Int. and impl
Duran [14]	To integrate user-centered design and implementation science principles to develop a virtual cardiac rehabilitation program	USA	1 hospital based hybrid home/clinic	HCPs, researchers, admin	Cardiac rehab-ilitation patients	Interviews, workshops, Obs, journey mapping, useability testing	QI project	User centred design- based Journey mapping	TDF and CFIR	Ap	SQUIRE	1. IS (TDF and CFIR) identified B&Fs to QI Int. impl 2. QI Int. designed and modified (journey mapping) 3. Pilot feasibility and useability tested using journey mapping
Farley [15]	To develop and implement a standardised post-fall debrief tool	USA	1 academic hospital	The QI project team (HCPs, clinical quality/risk manage-ment staff)	Fall patients	Survey, MRR, FGs, consensus workshops	QI initiative	PDSA, champions	REAIM	Ex	ND	1. QI tool designed and refined (PDSA) and implemented (Champions) 2. IS (REAIM) used to determine if appropriate to upscale intervention across org
Kallam [16]	To reduce sepsis in NICU through a hand hygiene QI project	Ghana	1 NICU in hospital	Hospital HCPs	NICU patients	Pre-post survey to test knowledge, Obs	QI initiative	LEAN methods, QI champions, process mapping, impact effort matrix	ISF	ND	ND	1. QI Int. developed and refined (Lean, champions, process mapping, impact effort matrix) 2. IS (ISF) guided impl. strategies
Kingsley [17]	Implement a screening tool to improve pain management referrals	USA	1 Paediatric hospital	Clinical psych-ologists and a social worker	Patients with sickle cell disease, 2–21 years	MRR	Impl. research	PDSA	CFIR	Ex	ND	1. IS (CFIR) informed the design QI tool drawing on B&Fs from feedback 2. Tool modified (PDSA) and implemented
Mathura [18]	Identify determinants that support the implementation of a BUN bundle to reduce laboratory testing	Canada	ED in 6 hospitals	ED physicians, nurse, unit clerk and medical learners*	ED patients	Interviews, and laboratory information system review, interrupted time series for cost estimations	Sequential explanatory mixed methods	Fish bone diagram, process mapping, PDSA, champ-ions	TDF, BCW, COM-B	Ap	The Good Reporting of a Mixed Methods Study	1. QI Int. developed and modified (fishbone diagram, process mapping, PDSA, champions) 2. IS (TDF and BCW/COM = B) used to identify B&Fs to QI impl. which then informed Impl. strategies
Middleton [19]	To evaluate the impact of the QI intervention on behavioural change of NICU staff, to improve SpO2 maintenance	ND (authors were based in Canada)	1 NICU within a hospital	NICU HCP staff	Sick and preterm infants	Pre and post questionnaire, audits and Obs of healthcare outcomes	A subset of a QI project	Cause and effect diagram. histogram event review reports, pareto chart analysis, Audit, Process mapping, force field analysis, PDSA	COM-B, TDF (incl. French's four steps)	Ex	ND	1. QI Int. designed and modified (Audit, cause and effect diagram, pareto chart analysis) 2. IS (COM-B and TDF) as well as Process mapping and force field analysis used to identify behaviour change techniques which informed QI Int. and impl. strategies 3. QI Int. refined (PDSA cycles)
Patel [20]	To develop and pilot a dashboard reporting individualised performance data for clinicians as a form of audit and feedback	USA	1 hospital	HCPs	ND	Workshops and surveys	Participatory design methodology	A&F -Cyclic adaptation based on feedback	COM-B	ND	ND	1. QI Int. developed (A&F) 2. IS (COM-B) identified impl. B&Fs which informed QI modification and impl. strategies
Silva [21]	To design and implement a care protocol for premature newborns in their first hour of life	Brazil	1 hospital with newborns	First line HCPs and managers	Babies and mothers	MRR, workshops, observation evaluation of training sessions, question-naire	A participatory research study	PDCA	CFIR	Ap	ND	1. QI Int. protocol developed (PDSA) 2. IS (CFIR) used to identify B&Fs to QI Int. impl, which informed modification of QI Int. and impl. strategies
Steinmo [22]	To implement a sepsis six bundle with support from behavioural science tools	UK	1 hospital	HCPs	Patients with sepsis	Interviews/FGs, Delphi process	A pragmatically designed QI programme	PDSA	TDF, BCT	Ex	TIDier	1. QI Int. designed and refined (PDSA) 2. IS (TDF and BCT) informed behaviour change techniques and implementation strategies 3. Modified QI impl. strategies finalised through Delphi exercise and consensus approach of APEASE criteria scores
Vanstone [23]	To improve the appropriate use of laboratory tests in the ED by implementing a clinician feedback dashboard	Can	ED in 2 hospitals	HCPs	ED patients	Laboratory information system data reviewed	QI project	PDSA, A&F, run charts, control chart analysis	COM-B/BCW	Ex	SQUIRE	1. IS (COM-B, BCW) informed QI Int. development (A&F dashboard) and QI Int. impl. strategies 2. Modifications made to QI Int. (PDSA) and impl. strategies 3. QI (Control chart and run chart analysis) used to measure changes pre- and post- intervention
Young [24]	To introduce an intradialytic exercise program	UK	Haemo-dialysis units in 2 hospitals	Physio-therapists	Patients on haemo-dialysis	Patient survey (incl. HADS, LUSS, DASI), audit, interviews, exercise records, MRR	QI project	3 PDSA cycles, Audit	TDF	Ex	SQUIRE	1. QI Int. developed (PDSA) 2. Second PDSA used IS (TDF) to identify B&Fs to QI Int. impl. which informed impl. strategies, along with A&F 3. QI Int. modified (PDSA)

*Abbreviations*:*ND* Not discussed, Country: ND not discussed (ND), *Can* Canada, *UK* United Kingdom, *USA* United States of America; Setting: *ED* Emergency Department, *NICU* Neonatal Intensive Care Unit; Participants: *admin* Administration, *HCPs* Health care Professionals, *not defined; Patients of QI Int. *Patients of QI intervention*; Data collection Methods: *DASI* The Dukes Activity Status Index, *FGs* Focus Groups, *HADS* The Hospital Anxiety and Depressions Scale, *LUSS* The Leicester Uraemic Symptoms Score, *MRR* Medical Record Review, *Obs* Observations; Study type: *impl* Implementation; QI tools/methods: *A&F* Audit and Feedback, *Lean* Lean six Sigma, *PDCA* Plan Do Check Act cycles, *PDSA* Plan Do Study Act cycles; IS theories: *BCW* Behaviour Change Wheel including Capabilities, *COM-B* Opportunity and Motivation, *BCT* The Behaviour Change Technique Taxonomy, *CFIR* The Consolidated Framework for Implementation Research, *ISF* The Interactive Systems Framework for Dissemination and Implementation, *TDF* Theoretical domains framework, and *RE-AIM* The Reach, Effectiveness, Adoption, Implementation, and Maintenance, framework; Ethics: *Ap* Approved, *Ex* Exempt, *ND* not discussed; Guideline followed for reporting: *TIDier* the Template for Intervention Development and Replication, *SQUIRE* Standards for Quality Improvement Reporting Excellence; Integration of QI and IS: *APEASE criteria* The affordability, practicability, effectiveness, acceptability, safety and equity criteria, *B&Fs* Barriers and facilitators to implementation, *impl* Implementation, *org* Organisation, *QI Int* QI intervention

#### Study participants

All of the studies involved healthcare professionals (HCPs), while some studies also included administrators [14], managers [21] and quality and risk management staff [15]. Studies included a mostly heterogenous set of patient cohorts with various health conditions: four studies included ‘hospitalised’ patients [13], including three studies of patients in the ED setting [18, 20, 23]; three studies included sick infants [16], including preterm infants [19] and babies and their mothers [21]; Other studies included cardiac rehabilitation patients [14]; fall patients [15]; children and young people with sickle cell disease [17]; patients with sepsis [22]; and patients on haemodialysis [24].

#### Study methods

The most commonly reported data collection methods were: surveys [13, 15, 16, 19–21, 24]; observations [13, 14, 16, 19, 21]; interviews and focus groups [13, 14, 18, 22, 24]; medical record and/or laboratory information system review [15, 18, 21, 23]; workshops [14, 20, 21]; and audits [19, 24] (Table 1).Of the 12 included studies, four reported receiving ethics, and six studies reported receiving ethics exemption. Only five reported using a reporting guideline [14, 18, 22–24] including three that reported using the Standards for Quality Improvement Reporting Excellence (SQUIRE) reporting guidelines [14, 23, 24], one that reported using the Template for Intervention Development and Replication (TIDier) reporting guidelines [22], and another that reported using the Good Reporting of a Mixed Methods Study [18] (Table 1).

### QI and IS components

**Fig. 2:**
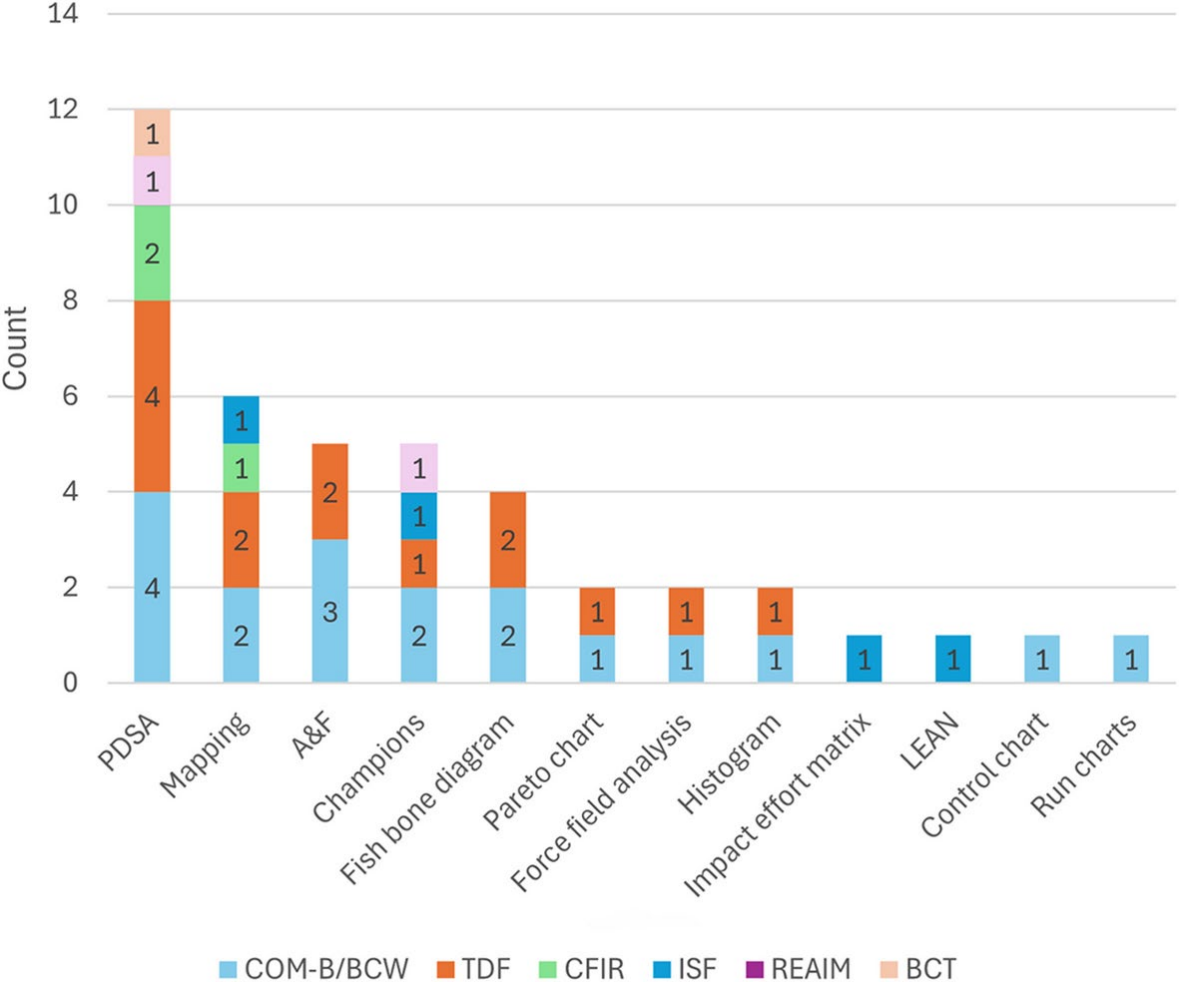
The frequency of reported IS frameworks used in conjunction with QI methods (Note: studies may be counted multiple times)

Of the 12 included studies, 12 key QI methods/tools were utilised including: Plan-Do-Study-Act (PDSA) cycles (*n* = 9), process mapping (*n* = 5), audit and feedback (A&F) (*n* = 5), QI champions (*n* = 4), fish bone diagram/cause and effect diagrams (*n* = 2), pareto charts (*n* = 1), force field analysis (*n* = 1), histograms (*n* = 1), impact effort matrix (*n* = 1), Lean six sigma (*n* = 1), control charts (*n* = 1) and run charts (*n* = 1) (Fig. 2, Table 1). Across the 12 included studies, the six IS theories and strategies used included: the Theoretical Domains Framework (TDF) (*n* = 5), Behaviour Change Wheel (BCW) including Capabilities, Opportunity and Motivation (COM-B) (*n* = 5), the Consolidated Framework for Implementation Research (CFIR) (*n* = 3), the Interactive Systems Framework for Dissemination and Implementation (ISF) (*n* = 1), the Reach, Effectiveness, Adoption, Implementation, and Maintenance (RE-AIM) (*n* = 1) framework, and the Behaviour Change Technique Taxonomy (BCT) (*n* = 1) (Fig. 2, Table 1). The most commonly paired IS and QI methods were the BCW/COM-B, TDF and CFIR used with PDSA, process mapping, and audit and feedback methods (Fig. 2).

### QI and IS integration

The narrative synthesis of studies identified that the process of QI and IS integration in the 12 studies typically followed one of two patterns: 1) IS theory/models were used to inform the initial development and design of the QI project in three studies [13, 17, 23]; 2) IS theory/models were used to inform the modification of the QI and QI implementation through the identification of determinants in 8 studies [13, 14, 17, 18, 20–22, 24]; or both [13, 17] (Table 1). A concise synopsis of the integration of QI and IS tools/theory developed from the narrative synthesis of studies can be seen in Table 1. These simplified steps highlight how QI and IS were utilised in each study.

**Fig. 3:**
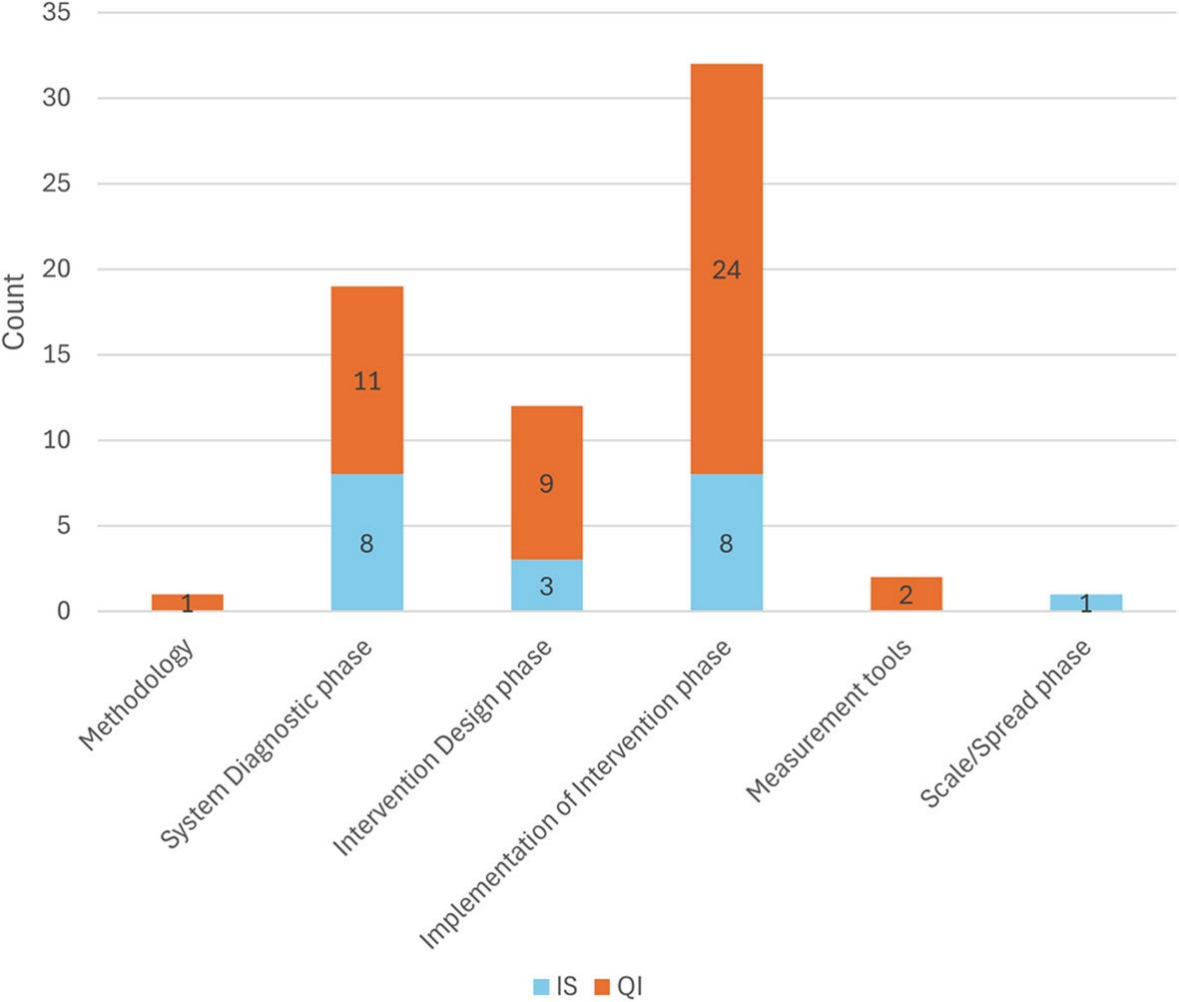
The frequency of use of QI/IS methods/tools/theory across study phases (Note: studies may be counted multiple times)

The key QI and IS methods/tools and theories used across the 12 included studies (see Table 1) were categorised into the six inductively identified phases of QI and IS studies. These included: *The System diagnostic phase* (which included process mapping, fishbone diagrams, pareto charts, force field analysis, impact effort matrices, histograms, BCW/COM-B, TDF, CFIR); *the Intervention design phase* (which included BCW/COM-B, CFIR); *the Implementation of intervention phase* which included intervention testing (PDSA) and embedded intervention strategies (audit and feedback, champions, BCW/COM-B, TDF, ISF, BCT); *the Scale/spread phase* (which included REAIM); *Methodology* (which included Lean Six Sigma); and Measurement tools (which included control charts and run charts). QI tools were used more in the System diagnostic, Intervention design and Implementation of intervention phases, however these three phases also utilised IS tools (Fig. 3).

## Discussion

This systematic review found 12 peer-reviewed studies that attempted to integrate QI and IS methods to implement a program in acute healthcare. The TDF, COM-B/BCW and CFIR were the most frequently used IS frameworks and the PDSA cycle was the most frequently used QI tool. As highlighted in Table 1, QI and IS methods were used sequentially or in parallel with one another, in a stepwise process to inform each stage of the study, however, no studies combined the methodologies, per se. The QI and IS methods/tools and theories were used in a distinct and independent manner across all of the included studies.

In addition to the 12 studies included in this review, the reasons for excluding studies during the full text review may provide some insight into how QI and IS are being used in health care. Of the 149 studies that underwent full text review, 65% (*n* = 97) were excluded because they described using QI or IS, however did not provide explicit descriptions or evidence of the use of individual frameworks or tools. This emphasises the lack of consistent reporting and terminology within and between the QI and IS fields. This definitional problem has been highlighted previously in reviews or commentaries comparing and contrasting the two fields [26, 27]. For the 12 studies that were included, the use of research methodological standards was the exception not the rule (*n* = 5, 42%), which may also contribute to the lack of consistent terminology. Similarly, there was inconsistent use of reporting guidelines to support the presentation of findings. These findings advocate for greater use of guidelines to enhance the rigour of QI and IS studies, as well as support more consistent terminology, through the use of the many guidelines currently available such as SQUIRE [28], the Standards for Reporting Implementation Studies (STARi) [29] or TIDier [30]. Agreed upon and harmonised definitions in both fields regarding concepts such as context, determinants, frameworks, strategies, and interventions will allow methods and results in studies to be more rigorously evaluated and learning to be shared [26].

Close to half of the studies at full text review stage were excluded (43%, *n* = 64/149) because they had used IS tools and theories only for “diagnostic” purposes or in other words, understanding the healthcare problem by identifying barriers and facilitators to implementation, rather than applying the findings to implement the intervention. An intervention applying these diagnostic findings may be reported in subsequent publications, but these were not identified by this review. This observation, that many studies use IS tools and theories solely for diagnostic purposes, aligns with previous findings from a systematic review on the use of the TDF to support healthcare clinician behaviour change; of the 60 studies in the review, just over half used the framework to inform barriers to, or to design implementation of interventions, but not undertake the intervention [31]. The observation also links to one of the key findings of our study: that in the different phases of implementation, there were differences in the use of QI and IS frameworks and tools. Whilst both QI and IS were used in the System diagnostic phase, and Intervention design phase, the Implementation phase tended to be dominated by the QI tool PDSA cycles. More guidance may be required on using IS frameworks to integrate tools from QI into implementation and evaluation. A number of prominent authors have highlighted that more integration of PDSA tools into IS studies is warranted [26].

The choice of IS frameworks used in the 12 included studies may assist in explaining the variable application of IS in these studies. Of the 16 instances of IS frameworks used in our 12 included studies, 81% (*n* = 13/16) utilised the COM-B/BCW, TDR, or CFIR. In Nilsen’s model of IS implementation theories, models and frameworks [26], these three are all used to assist with understanding or explaining what influences implementation outcomes. They do not assist with describing and/or guiding the process of translating research into practice, like the Knowledge to Action framework [32]. In other words, they are providing frameworks of what to do, rather than providing a mechanism to test the strategies and to respond or make changes. Greater guidance is needed to support the use of flexible IS methods and theories that can support rapid implementation of improvements within the context of a complex adaptive system such as healthcare [33].

Similarly, calls have been made to provide more theory to QI studies [34]. The results of our study bear this out where 3/12 studies used IS frameworks to inform the design phase. Designing interventions using both informal and formal theories supports the analysis and description of the rationale and assumptions about mechanism of actions, and the link between processes to outcomes [34]. In turn, they can inform an evaluation framework.

Overall, the review identified some integration of QI and IS across design, system diagnostic and implementation phases, however the domains of spread, reach and sustainability require further work. There was also minimal discussion of the impact of integration of QI and IS in the included studies.

### Strengths and limitations

A strength of the review was the adherence to an international standard of systematic review methodology (PRISMA). Five databases were searched to maximise the opportunity for studies to be included. The reviewers were all experienced in the fields of IS and QI methods.

There are several limitations to this review. Firstly, the included IS studies tended to use the COM-B and CFIR frameworks, however this was largely due to the use of those terms in the search string, which was not exhaustive. This was underpinned by an assumption that the term “implementation science” would yield studies using a broad range of frameworks. Future analysis using search terms reflecting other IS frameworks may be useful to enhance these findings. Another limitation of the review was that agreement between reviewers on which studies to include was variable. This reflected two issues: that definitions for QI and IS studies are not harmonised; and that studies may state that they fit under an IS or QI banner, but they do not necessarily explicitly describe the respective tools. To mitigate this low Kappa score, all disagreements were discussed as a team, and consensus reached as to whether a manuscript would be included or excluded, and why. This review was also limited to studies set in a tertiary hospital setting, and published since 2014, limiting a comparison to other settings and to older literature. The review only included studies that clearly demonstrated and explained the QI and IS tools used, meaning that studies that did not explain their use of QI or IS clearly were excluded. The review also only included studies published in English.

## Conclusion and implications for future research

QI and IS methodologies have been developed independently over time, but this review has identified studies where the integration of the two approaches has been attempted. To encourage further integration of QI and IS, greater guidance is needed on the best approach to the harmonisation of existing frameworks and the use of consistent terminology. These actions would help to move researchers beyond the diagnostic role often taken and encourage theory informed action. There is a clear need for research guidance on how and when to select, justify, and integrate appropriate QI and IS methods and theory within healthcare studies, supported by greater use of reporting guidelines in QI and IS studies, to enhance overall implementation and sustainability of improvement projects.

## Supplementary Information

Supplementary Material 1. PRISMA 2020 checklist.

Supplementary Material 2. Medline Search terms.

Supplementary Material 3. Explanation and examples of the analysis for Fig. 3.

Supplementary Material 4. Critical appraisal of studies using the QI-MQCS tool.

## Data Availability

The datasets used and/or analysed during the current study are available from the corresponding author on reasonable request.

## Abbreviations

BCT: Behaviour Change Technique Taxonomy
BCW: Behaviour Change Wheel
BUN: Blood Urea Nitrogen
CFIR: Consolidated Framework for Implementation Research
ED: Emergency Department
HCPs: Healthcare professionals
IS: Implementation Science
ISF: Interactive Systems Framework for Dissemination and Implementation
NICU: Neonatal Intensive Care Unit
PDSA: Plan-Do-Study-Act
PRISMA: Preferred Reporting Items for Systematic Reviews and Meta- Analyses
QI: Quality Improvement
QI-MQCS: QI Minimum Quality Criteria Set
RE-AIM: Reach, Effectiveness, Adoption, Implementation, and Maintenance
SQUIRE: Standards for Quality Improvement Reporting Excellence
TIDier: Template for Intervention Development and Replication
TDF: Theoretical Domains Framework
UK: United Kingdom
USA: United States of America
