# Supplementary material for: The integration of quality improvement and implementation science methods and frameworks in healthcare: a systematic review
Source: BMC Health Serv Res. 2025 Apr 16;25:558. doi: 10.1186/s12913-025-12730-9 (PMC12001488; doi:10.1186/s12913-025-12730-9)
Supplement: Supplementary file 2 — Supplementary Material 2. Medline Search terms. [file 12913_2025_12730_MOESM2_ESM.pdf]

Supplementary file 2. Medline Search terms
Ovid MEDLINE(R) ALL <1946 to June 17, 2024>

1	exp Quality Improvement/	35165
2	Quality Improvement.ti,ab,kf.	58296
3	exp Total Quality Management/	12772
4	"total quality management".ti,ab,kf.	1096
5	Continuous Improvement.ti,ab,kf.	3765
6	Improvement science.ti,ab,kf.	265
7	lean methodology.ti,ab,kf.	200
8	Lean management.ti,ab,kf.	297
9	Plan-Do-Study-Act cycle.ti,ab,kf.	206
10	PDSA.ti,ab,kf.	1314
11	exp "Root Cause Analysis"/	445
12	"Root cause analys*".ti,ab,kf.	1665
13	Kaizen.ti,ab,kf.	147
14	RCA.ti,ab,kf.	8150
15	Six sigma.ti,ab,kf.	967
16	six sigma methodology.ti,ab,kf.	166
17	"Institute for Healthcare Improvement Model for Improvement".ti,ab,kf.	26
18	"Theory of constraint*".ti,ab,kf.	85
19	exp Implementation Science/ or exp "diffusion of innovation"/ or ("The Consolidated Framework for Implementation Research" or "Theoretical domains framework" or "Reach effectiveness adoption implementation Maintenance" or "RE-AIM" or "The Knowledge-to-Action Framework" or "Diffusion of Innovation* Theory" or "Implementation climate scale" or "Com-b" or "reach, effectiveness, adoption, implementation, and maintenance framework").ti,ab,kf.	27723
20	exp Quality Improvement/ or total quality management/ or exp "Root Cause Analysis"/ or ("Quality Improvement" or "total quality management" or "Continuous Improvement" or "Improvement science" or "lean methodology" or "Lean management" or "Plan-Do-Study-Act cycle" or "PDSA" or RCA or "Root cause analys*" or Kaizen or "Six sigma" or "six sigma methodology" or "Institute for Healthcare Improvement Model for Improvement" or "Theory of constraint*").ti,ab,kf.	100391
21	exp implementation science/	1473
22	"The Consolidated Framework for Implementation Research".ti,ab,kf.	1931
23	"Theoretical domains framework".ti,ab,kf.	1554
24	"Reach effectiveness adoption implementation Maintenance".ti,ab,kf.	267
25	RE-AIM.ti,ab,kf.	1507
26	"The Knowledge-to-Action Framework".ti,ab,kf.	145
27	exp "diffusion of innovation"/	21777
28	"Diffusion of Innovation* Theory".ti,ab,kf.	459
29	"Implementation climate scale".ti,ab,kf.	25
30	Com-b.ti,ab,kf.	987
31	"reach, effectiveness, adoption, implementation, and maintenance framework".ti,ab,kf.	283
32	"Delivery of Health Care"/	123133
33	health care/	123133
34	"health care".ti,ab,kf.	481534
35	healthcare.ti,ab,kf.	388765
36	1 or 2 or 3 or 4 or 5 or 6 or 7 or 8 or 9 or 10 or 11 or 12 or 13 or 14 or 15 or 16 or 17 or 18	100391
37	32 or 33 or 34 or 35	886307
38	21 or 22 or 23 or 24 or 25 or 26 or 27 or 28 or 29 or 30 or 31	27723
39	36 and 37 and 38	609
40	36 and 38	1384

Note with Web of Science search, an additional "health care" search term was included, as per advice from a librarian.
