# Supplementary material for: The integration of quality improvement and implementation science methods and frameworks in healthcare: a systematic review
Source: BMC Health Serv Res. 2025 Apr 16;25:558. doi: 10.1186/s12913-025-12730-9 (PMC12001488; doi:10.1186/s12913-025-12730-9)
Supplement: Supplementary file 3 — Supplementary Material 3. Explanation and examples of the analysis for Fig. 3. [file 12913_2025_12730_MOESM3_ESM.pdf]

Supplementary file 3. Inductive narrative analysis that identified key study phases, figure 3

The narrative analysis of the integration of QI and IS in the studies in conjunction with frequency analysis was used to identify how frequently QI or IS methods/tools/theories were used across different phases of QI and IS studies. Studies were analysed line-by-line to identify the QI or IS methods/tools/theories used (See table 1, Characteristics of studies). Key study phases were inductively identified during the narrative analysis, into which the methods/tools/theories could be categorised:

- *The System diagnostic phase*, which we defined as an assessment of the extent and/or nature of an issue being targeted to improve performance or outcomes, and identification of barriers and facilitators (B&Fs) to implementation. This included: QI methods/tools/theories used to identify B&Fs to implementation (e.g., Process Mapping, Fishbone diagram/ Cause and effect diagram, Pareto chart, Force field analysis, Impact effort matrix, and histograms), and IS tools/theories used to identify B&Fs to implementation (e.g., COM-B, TDF, CFIR).
- *The Intervention design phase* which typically involves the design, development and refinement of an intervention. This included: QI/IS methods/tools/theories used to inform the QI design.
- *The Implementation of intervention phase* which typically included intervention testing and embedded strategies to implement the intervention. This included: QI tools/findings that guided implementation strategies (e.g., Plan, Do, Study, Act (PDSA), Audit and & Feedback (A&F), and Champions), IS tools/theories that guided implementation strategies, and Feasibility and useability testing.
- The *Scale/spread or sustainability phase* which included scale up of the intervention to a larger or different team or setting with consideration of ongoing maintenance of the implementation of the intervention. This included IS tools/theories used to determine whether it was appropriate to upscale the intervention across the organization.
- As well as these four phases, ***Methodology*** (which included methodologies that were applied across the entire span of the study such as Lean six sigma), and ***Measurement tools*** such as Control charts and Run charts were included in the analysis.

Phase	Tools/methods/theories
Systems Diagnostic phase	Process Mapping
	Fishbone diagram (Cause and effect diagram)
	Pareto chart
	Force field analysis
	Impact effort matrix
	Histogram
	IS used to identify B&Fs
Intervention Design phase	IS/ QI informed QI design
Implementation of intervention phase	PDSA
	A&F
	Champions
	IS/ QI guided implementation strategies
	Intervention feasibility and useability testing
Scale/spread phase	IS used to determine if it is appropriate to upscale the intervention across the organisation
Measurement tools	Control chart
	Run charts
Methodology	LEAN

Example of analysis

QI and IS study phases	IS tools/theory used	QI tools/methods used
**Systems Diagnostic phase**		
Process Mapping		Cummings 2017<br>Duran 2023<br>Kallam 2018<br>Mathura 2023<br>Middleton 2022
Fishbone diagram (Cause and effect diagram)		Mathura 2023<br>Middleton 2022
Pareto chart		Middleton 2022
Force field analysis		Middleton 2022
Impact effort matrix		Kallam 2018
Histogram		Middleton 2022
IS used to identify B&Fs	Cummings 2017<br>Duran 2023<br>Kingsley 2020<br>Mathura 2023<br>Patel 2022<br>Silva 2023<br>Steinmo 2016<br>Young 2018	
**Intervention Design phase**		
IS/ QI informed QI design	Cummings 2017<br>Kingsley 2020<br>Vanstone 2022	Duran 2023<br>Farley 2023<br>Kallam 2018<br>Middleton 2022<br>Mathura 2023<br>Patel 2022<br>Silva 2023<br>Steinmo 2016<br>Young 2018
**Implementation of intervention phase**		
PDSA		Cummings 2017<br>Farley 2023<br>Kingsley 2020<br>Mathura 2023<br>Middleton 2022<br>Silva 2023 (PDCA)<br>Steinmo 2016<br>Vanstone 2022<br>Young 2018
A&F		Cummings 2017<br>Middleton 2022<br>Patel 2022<br>Vanstone 2022<br>Young 2018
Champions		Cummings 2017<br>Farley 2023<br>Kallam 2018<br>Mathura 2023
IS/QI guided implementation strategies	Kallam 2018<br>Mathura 2023<br>Middleton 2022<br>Patel 2022	Cummings 2017<br>Kingsley 2020<br>Middleton 2022

	Silva 2023<br>Steinmo 2016<br>Vanstone 2022<br>Young 2018	Vanstone 2022<br>Young 2018
Feasibility and useability testing		Duran 2023
**Scale/spread phase**		
IS used to determine if it is appropriate to upscale the intervention across the organisation	Farley 2023	
**Measurement tools**		
Control chart		Vanstone 2022
Run charts		Vanstone 2022
**Methodology**		
LEAN		Kallam 2018
