# Supplementary material for: The integration of quality improvement and implementation science methods and frameworks in healthcare: a systematic review
Source: BMC Health Serv Res. 2025 Apr 16;25:558. doi: 10.1186/s12913-025-12730-9 (PMC12001488; doi:10.1186/s12913-025-12730-9)
Supplement: Supplementary file 4 — Supplementary Material 4. Critical appraisal of studies using the QI-MQCS tool. [file 12913_2025_12730_MOESM4_ESM.pdf]

Supplementary file 4. Critical appraisal of studies using the QI-MQCS tool

Study	Cummings 2017	Duran 2023	Farley 2023	Kallam 2018	Kingsley 2020	Mathura 2023	Middleton 2022	Patel 2022	Silva 2023	Steinmo 2016	Vanstone 2022	Young 2018
QI-MQCS Critical appraisal domain (not met: NM, Met, M, scoring 1 or 0)												
Organizational Motivation: Names or describes at least one motivation for the organization's participation in the intervention	M	M	M	M	M	M	M	M	M	M	M	M
Intervention Rationale: Names or describes a rationale linking at least one central intervention component to intended effects	M	M	M	M	M	NM	M	M	M	M	M	M
Intervention Description: Describes at least one specific change in detail including the personnel executing the intervention	M	M	M	M	M	M	M	M	M	M	M	M
Organizational Characteristics: Reports at least two organizational characteristics	M	NM	M	M	M	M	M	M	M	NM	M	M
Implementation: Names at least one approach used to introduce the intervention	M	M	M	M	M	M	M	M	M	M	M	M
Study Design: Names the study design	M	M	NM	NM	NM	M	NM	M	NM	NM	NM	NM
Comparator: Describes at least one key care process	NM	M	NM	M	NM	M	M	NM	M	NM	M	M
Data Source: Describes the data source and defines the outcome of interest	M	M	M	M	M	M	M	M	M	M	M	M
Timing: Describes the timing of the intervention and evaluation to determine the presence of baseline data and the followup period after all intervention components were fully implemented	M	M	M	M	M	M	M	M	M	M	M	M
Adherence / Fidelity: Reports fidelity information for at least one intervention component, or describes evidence of adherence or a mechanism ensuring compliance to the intervention	M	NM	M	M	M	NM	M	M	NM	NM	M	M
Health Outcomes: Reports data on at least one health-related outcome	M	NM	NM	NM	NM	NM	NM	NM	NM	M	NM	M
Organizational Readiness: Reports at least one organizational-level barrier or facilitator	M	M	NM	M	M	M	NM	M	M	M	M	M
Penetration / Reach: Describes the proportion of all eligible units who actually participated	M	M	NM	NM	M	M	NM	M	M	NM	NM	M
Sustainability: Describes the sustainability or the potential for sustainability	M	M	M	NM	NM	NM	M	M	M	NM	NM	M
Spread: Describes the potential for spread, existing tools for spread, or spread attempts / largescale rollout	NM	NM	M	NM	NM	NM	M	M	NM	NM	NM	NM
Limitations: Reports at least one limitation of the design / evaluation	M	M	M	M	M	M	M	M	M	M	M	M
Score out of 16	14	12	11	11	11	11	12	14	12	9	11	14
